# *ACTN3* Genotype, Athletic Status, and Life Course Physical Capability: Meta-Analysis of the Published Literature and Findings from Nine Studies

**DOI:** 10.1002/humu.21526

**Published:** 2011-05-03

**Authors:** Tamuno Alfred, Yoav Ben-Shlomo, Rachel Cooper, Rebecca Hardy, Cyrus Cooper, Ian J Deary, David Gunnell, Sarah E Harris, Meena Kumari, Richard M Martin, Colin N Moran, Yannis P Pitsiladis, Susan M Ring, Avan Aihie Sayer, George Davey Smith, John M Starr, Diana Kuh, Ian NM Day, the HALCyon study team

**Affiliations:** 1 School of Social and Community Medicine, University of Bristol, Bristol, United KingdomLondon, United Kingdom; 2 MRC Unit for Lifelong Health and Ageing and Division of Population Health, University College LondonLondon, United Kingdom; 3 MRC Lifecourse Epidemiology Unit, University of SouthamptonSouthampton, United Kingdom; 4 Institute of Musculoskeletal Sciences, University of OxfordOxford, United Kingdom; 5 Centre for Cognitive Ageing and Cognitive Epidemiology, University of EdinburghEdinburgh, United Kingdom; 6 Department of Psychology, University of EdinburghEdinburgh, United Kingdom; 7 Medical Genetics Section, University of EdinburghEdinburgh, United Kingdom; 8 Department of Epidemiology and Public Health, University College LondonLondon, United Kingdom; 9 MRC Centre for Causal Analyses in Translational Epidemiology, University of BristolBristol, United Kingdom; 10 Centre for Systems Biology, University of EdinburghEdinburgh, United Kingdom; 11 Institute of Cardiovascular and Medical Sciences, College of Medical, Veterinary and Life Sciences, University of GlasgowGlasgow, United Kingdom; 12 Academic Geriatric Medicine, University of Southampton, Southampton General HospitalSouthampton, United Kingdom; 13 Geriatric Medicine Unit, University of Edinburgh, Royal Victoria HospitalEdinburgh, United Kingdom

**Keywords:** *ACTN3*, Actinin-3, athlete, aging, SNP, grip strength

## Abstract

The *ACTN3* R577X (rs1815739) genotype has been associated with athletic status and muscle phenotypes, although not consistently. Our objective was to conduct a meta-analysis of the published literature on athletic status and investigate its associations with physical capability in several new population-based studies. Relevant data were extracted from studies in the literature, comparing genotype frequencies between controls and sprint/power and endurance athletes. For life course physical capability, data were used from two studies of adolescents and seven studies in the Healthy Ageing across the Life Course (HALCyon) collaborative research program, involving individuals aged between 53 and 90+ years. We found evidence from the published literature to support the hypothesis that in Europeans the RR genotype is more common among sprint/power athletes compared with their controls. There is currently no evidence that the X allele is advantageous to endurance athleticism. We found no association between R577X and grip strength (*P* = 0.09, *n* = 7,672 in males; *P* = 0.90, *n* = 7,839 in females), standing balance, timed get up and go, or chair rises in our studies of physical capability. The *ACTN3* R577X genotype is associated with sprint/power athletic status in Europeans, but does not appear to be associated with objective measures of physical capability in the general population. Hum Mutat 32:1–11, 2011. © 2011 Wiley-Liss, Inc.

## Introduction

Genetic association studies have identified several loci associated with physical performance phenotypes [Bray et al., 2009]. One of the more common investigations has been into a single nucleotide polymorphism (SNP) in the alpha-actinin-3 (*ACTN3*; MIM# 102574) gene. *ACTN3* is expressed in fast twitch muscle fibers [North et al., 1999], the fiber types that contract quickly but are less resistant to fatigue [Allen et al., 2008]. SNP rs1815739 (R577X) encodes a premature stop codon leaving individuals with two copies of the T allele (XX homozygotes) completely deficient in the protein [North et al., 1999]. There is, however, no evidence for associations with disease phenotypes [North et al., 1999; Rubio et al., 2007]. However, studies investigating R577X genotype frequencies in athletes and the general population have found that its C (R) allele is overrepresented in sprinters or power athletes compared with controls [Eynon et al., 2009b; Papadimitriou et al., 2008; Yang et al., 2003] or endurance athletes [Eynon et al., 2009b; Niemi and Majamaa, 2005; Yang et al., 2003]. Other reports show its overrepresentation in professional soccer players [Santiago et al., 2008], artistic gymnasts [Massidda et al., 2009], endurance [Ahmetov et al., 2008], strength- and power-oriented athletes [Druzhevskaya et al., 2008; Roth et al., 2008] compared with the general population. In addition, the *ACTN3* R577X genotype may also influence sprint performance in combination with other genotypes [Eynon et al., 2009a, 2010]. However, many studies have not found differences in the R577X genotype frequencies between endurance athletes and controls [Doring et al., 2010; Muniesa et al., 2008; Paparini et al., 2007; Saunders et al., 2007; Yang et al., 2007] or between sprint/power athletes and controls [Scott et al., 2010; Yang et al., 2007], and no summary meta-analysis has been presented.

Genotypes associated with athletic status may also contribute to the interindividual variability in physical capability, the capacity to undertake the physical tasks of daily living, through effects on muscle function or maintenance. Physical capability declines from midlife onward [Himann et al., 1988; Mathiowetz et al., 1985] and within specific age groups lower levels of physical capability, as assessed by objective measures including grip strength and standing balance, have been associated with poorer cognition [Coppin et al., 2006; Deary et al., 2006; Kuh et al., 2009] and are predictors of increased morbidity [Cooper et al., 2011; Guralnik et al., 1995; Ortega et al., 2008] and mortality rate [Cooper et al., 2010]. Twin studies have shown that measures of physical capability are partly heritable [Arden and Spector, 1997; Carmelli et al., 2000; Tiainen et al., 2004]; for example, in older females the genetic component has been shown to explain 14% of grip strength variability [Tiainen et al., 2004]. Therefore, it has been hypothesized that the *ACTN3* R577X genotype may be associated with measures of physical capability. In the general population, one investigation suggested associations between R577X and decline in walk times in older men and persistent lower extremity limitation in older women [Delmonico et al., 2008]. Another study [Moran et al., 2007] observed that the R allele was associated with faster sprint times in adolescent males, but not in females, whereas another study of young adults showed no effect [Santiago et al., 2009]. Sex differences have also been reported regarding associations with weight [Delmonico et al., 2008; Walsh et al., 2008], body mass index (BMI) [Walsh et al., 2008], and physical activity levels [Delmonico et al., 2008].

We conducted a systematic review and meta-analysis of the reports on the relationship between the *ACTN3* R577X genotype and athletic status. We also examined associations between *ACTN3* R577X and physical capability phenotypes in seven UK cohorts of middle-aged and older adults as part of the HALCyon (Healthy Aging across the Life Course; http://www.halcyon.ac.uk) collaborative research program, one cohort with measures at age 11 years [Golding et al., 2001], and one previously reported cross-sectional study of adolescents [Moran et al., 2007]. We also investigated anthropometric traits, which have been shown to be associated with physical capability [Kuh et al., 2005b; Samson et al., 2000]. To our knowledge, this is the first report of a meta-analysis of the *ACTN3* R577X genotype and athletic status and by far the largest investigation into its association with physical capability in the general population.

## Materials and Methods

### Literature Search on Athletic Status

A search of electronic databases was conducted to identify all publications on *ACTN3* and athletic status up to November 29, 2010. The search terms “ACTN3” and “actinin-3” were used, with no restrictions to date or language, in Medline and Web of Science producing 298 hits, of which 187 were duplicates. Of the 111 unique publications, 21 studies presented data on athletes and nonathletes, although two were from one group of authors reporting on the interactions between *ACTN3* R577X and other genotypes, having previously reported on the *ACTN3* R577X genotype alone, and six were from one group of authors who appeared to repeat data on the groups of controls and endurance athletes; therefore, the publication reporting on both athletic groups and using the largest numbers is cited here. From the remaining 14 publications, the numbers of RR, RX, and XX individuals in the controls and athletic groups by ancestral group were extracted or estimated from the given percentages from 13 publications. Where available in the publication, this was also done by sex. Attempts were not successful to trace the contact details of the authors of the one study [Saunders et al., 2007] for which genotype frequencies were not given in the article. Additional details extracted from the articles that were not included in the meta-analysis but may be informative about characteristics of the study participants include the sports or events of the athletic groups and the source of controls. The reporting of the Hardy-Weinberg equilibrium (HWE) test was checked in all articles, although no exclusions were made if departure was reported in a control group. A flow diagram of the identification of the studies is presented in Supp. Figure S1.

### Physical Capability Studies

The Avon Longitudinal Study of Parents and Children (ALSPAC) comprises 14,541 pregnancies with estimated due dates between April 1, 1991 and December 31, 1992, in Avon, England. We used measurements and tests taken at 11 years (between 2003 and 2005), when all children were invited to a Focus Clinic Session. Blood samples from the children have been collected since birth. Ethical approval for the study was obtained from the ALSPAC Law and Ethics Committee and Local Research Ethics Committees. Further details of the study are available [Golding et al., 2001].

Eureka is a cross-sectional study of 1,198 healthy 11- to 18-year-olds recruited from 10 rural and urban schools around Trikala, Greece. Measures of body composition, strength/power and endurance phenotypes were taken and DNA was extracted from buccal cell samples. Details of the study have been previously described [Moran et al., 2006, 2007].

The Medical Research Council National Survey of Health and Development (NSHD) comprises participants sampled from all births in a week in March 1946 and followed up since. In 1999, at age 53 years, men and women were visited by a research nurse and consent for DNA extraction was given by approximately 2,900 members of the cohort. Details of the data collected and the several phases of the study are available on the cohort's Website (http://www.nshd.mrc.ac.uk) and elsewhere [Wadsworth et al., 2006].

The English Longitudinal Study of Ageing (ELSA) comprises men and women aged 50 years and over who originally participated in the Health Survey for England in 1998, 1999, or 2001. Fieldwork began in 2002–2003 (Phase I) with two yearly follow-ups and in 2004–2005 (Phase II) blood samples were provided by 6,231 participants. Details of the cohort have been published [Marmot et al., 2003].

The Hertfordshire Cohort Study (HCS) consists of 2,997 participants born 1931–1939 and registered with a General Practitioner in East, North, and West Hertfordshire who attended a clinic in 1994–2004 (Phase I). A second assessment took place in 2004–2005 for participants in East Hertfordshire (Phase II). Further details of study design, data collected, and summaries of participant characteristics have been published [Syddall, 2005].

The Hertfordshire Ageing Study (HAS) comprises men and women traced in 1994–1995, the first follow-up (Phase I), of singleton births from 1920–1930 in North Hertfordshire. A total of 717 participants attended a clinic during which DNA was extracted. A second follow-up took place in 2003–2005 (Phase II). Details of the recruitment, data collected, and summaries of participant characteristics have been described previously [Syddall et al., 2009].

The Boyd Orr cohort is a historical cohort study based on children surveyed in 1937–1939 in English and Scottish districts. Participants were followed up in 1997–1998 (Phase II) and again in 2002–2003 (Phase III), during which DNA was extracted from 728 adults. Details of the study design and the data collected have been described on its Website (http://www.epi.bris.ac.uk/boydorr) and elsewhere [Martin et al., 2005].

The Caerphilly Prospective Study (CaPS) recruited 2,512 men aged between 45 and 59 years in 1979–1983 from the town of Caerphilly, South Wales, and its surrounding villages. Blood samples were collected at baseline and at each of the four follow-ups (Phase II: 1984–1988, Phase III: 1989–1993, Phase IV: 1993–1997, and Phase V: 2002–2004.) Further details are available on the cohort's Website (http://www.epi.bris.ac.uk/caerphilly/caerphillyprospectivestudy.htm).

The Lothian Birth Cohort 1921 Study (LBC1921) participants were all born in 1921 and completed an IQ assessment age 11. In 1999–2001 (Phase I) 550 79-year-olds, living in and around Edinburgh, attended a clinic, and in 2003–2005 (Phase II) 321 returned at 83 years old. Details of the recruitment into the study are available on its Website (http://www.lothianbirthcohort.ed.ac.uk) and have been published previously [Deary et al., 2004; Gow et al., 2008].

### Genotyping and Quality Control

Genotyping for *ACTN3* SNP rs1815739 (R577X) for all studies, except LBC1921, ALSPAC and Eureka, was carried out by KBioscience (http://www.kbioscience.co.uk). Genotype information in LBC1921 and ALSPAC came from genome-wide scans performed on the Illumina (http://www.illumina.com) Human610-Quadv1 Chip and Human-Hap317K BeadChip, respectively [Houlihan et al., 2010; Timpson et al., 2009]. In Eureka, genotypes were assessed using the TaqMan SNP Assay C_590093_1 (Applied Biosystems, Melbourne, Australia; http://www.appliedbiosystems.com.au) with around 10% of genotypes validated using RFLP or direct sequencing. Data quality was reviewed by assessing departure from HWE, clustering quality (using KBioscience software SNPviewer on their data) and call rates.

### Phenotypes

#### Categorization of athletic status in literature

Descriptions of the sports or events among the athletic groups, as given in the publications, as well as their classification into either sprint/power or endurance status, for the studies included in the meta-analysis, are presented in Supp. Table S1.

#### Anthropometry

Measurements were conducted either at clinics, during a clinical interview in the home, from self-report, or, in Eureka, during physical education classes. BMI (kg/m^2^) was calculated as weight divided by height squared. Waist-hip ratio (WHR) was defined as waist circumference (cm) divided by hip circumference (cm) and was measured in ALSPAC, NSHD, ELSA, HCS, HAS, Boyd Orr, and CaPS.

#### Physical capability and activity

The physical capability measures taken in the different studies are listed in Table 1. Grip strength was measured using electronic or hydraulic dynamometers, with the best measure used in the analysis where more than one trial was conducted. Standing balance tests were conducted in the studies, with participants' eyes open: flamingo [Committee of Experts on Sports Research, 1993] (stopped at 30 sec) and side-by-side, semitandem and full tandem [Stevens et al., 2008]. Poor standing balance was defined for this analysis as the inability to complete 30 sec, or 5 sec of the full tandem. The timed get up and go test [Podsiadlo and Richardson, 1991] required participants to get up from a chair, walk 3 m, turn, walk back, turn, and sit down. Timed walks over 2.44 m (8 feet) and 6 m were carried out with the fastest time used in the analysis where more than one trial was conducted. Natural log transformations were used to improve the normality of timed walks and get up and go. Timed chair rises [Csuka and McCarty, 1985] involved asking participants to rise from a chair and sit back down 5 or 10 times; the reciprocal of time taken in seconds × 100 [Kuh et al., 2005a] was used in the analysis. Levels of physical activity were derived from self reports levels using questionnaires.

**Table 1:** Summary of Sex, Age, and *ACTN3* R577X Minor Allele Frequencies by Cohort

Cohort	Age^a^ in years, median (range)	Male, %	MAF	Total	Physical capability measures included in present analysis
ALSPAC	11 (10–13)	51	0.44	2,967	Grip strength
Eureka	15 (11–18)	53	0.42	992	Grip strength
NSHD	53	50	0.44	2,595	Grip strength, standing balance,^b^ timed chair rises
ELSA	65 (52–90+)	46	0.44	5,435	Grip strength, standing balance,^b^ timed walk,^c^ timed chair rises^d^
HCS	66 (59–73)	53	0.43	2,831	Grip strength, standing balance,^b^ TGUG, timed chair rises^d^
HAS	67 (63–73)	61	0.42	508	Grip strength, TGUG, timed chair rises^d^
Boyd Orr	70 (64–82)	46	0.46	684	Standing balance,^b^ TGUG
CaPS	73 (65–83)	100	0.42	1,309	Standing balance,^b^ TGUG
LBC1921	79 (77–80)	41	0.45	514	Grip strength, timed walk^c^
Total	60 (10–90+)	53	0.43	17,835	

a Age at phase from which the majority of variables are taken.

b Flamingo in NSHD, HCS, HAS, Boyd Orr, and CaPS; side-by-side, semitandem, and full tandem in ELSA.

c 2.44 m (8 feet) in ELSA, 6 m in LBC1921.

d Five rises in ELSA, HCS, and HAS, 10 rises in NSHD. Genotype frequencies by sex presented in Suppl. Table S2. Minor allele: X. TGUG, timed get up and go.

### Statistical Methods

Statistical analysis was performed in Stata 11.1 (StataCorp LP). A two-tailed significance level of *P*<0.05 was used. Reporting of the meta-analyses met the appropriate items of a recommended checklist [Stroup et al., 2000].

#### Studies of athletic status

Fixed and random-effects meta-analyses [Egger et al., 1997a] using inverse-variance weighting were used to combine results from the studies on athletic status, using odds ratios, for the following comparisons: sprint/power versus controls, endurance versus controls, and sprint/power versus endurance. Due to a number of studies reporting no XX individuals among sprint/power athletes [Massidda et al., 2009; Roth et al., 2008; Yang et al., 2003, 2007] or among their respective controls [Yang et al., 2007], a dominant model for the X allele (RR vs. RX + XX) was used in the analysis for sprint/power versus controls or endurance, whereas both a dominant and recessive (RR + RX vs. XX) model were used in endurance versus controls. Data were stratified by ancestral group and, where data were reported separately in males or females, by sex. To investigate publication bias, or more broadly, “small study effects,” funnel plots, and the Egger test were used [Egger et al., 1997a, b]. Heterogeneity was investigated, with the I^2^ measure used for its quantification [Higgins et al., 2003].

#### Studies of physical capability

Where information on ancestry was collected, non-European participants were excluded from the analyses in order to avoid confounding from population stratification [Cordell and Clayton, 2005]. Within studies, linear and logistic regression analyses were conducted on the continuous and dichotomous traits within the cohorts, respectively. Additive models were used with genotypes coded as 0, 1, and 2 for the number of X alleles. Likelihood ratio tests were used to compare the fit of the additive models compared with the full genotype model. For continuous traits, the normality of the standardized residuals was inspected with distributional diagnostic plots. Cook's distances [Cook, 2000] were plotted against fitted values, using a cutoff of four divided by sample size, to identify influential outliers in the continuous phenotypes. For the harmonization of continuous traits that were used to obtain pooled estimates of the genotypic effects, *z*-score units were calculated in each study by subtracting the study mean and dividing by its standard deviation, using the data on the individuals included in the present analysis only. The overall mean for *z*-scores is 0 and standard deviation 1. Beta coefficients calculated on *z*-score units can be reverted to the original scale by multiplying by an appropriate standard deviation. Two-step [Riley et al., 2010] meta-analyses were performed to obtain pooled genotypic effects, with the *P*-values from the random-effects model presented in the tables. The I^2^ measure was used to quantify heterogeneity. Due to the different genotypic effects reported (Introduction), *z*-scores were calculated and analyses were performed separately in males and females. In addition, analyses were also stratified by physical activity, an indicator shown to modify genotypic effects on anthropometric measures [Li et al., 2010]. Within-study investigations were also made into follow-up measures of grip strength adjusting for the measure in an earlier phase in HCS, HAS, and LBC1921, due to its collection in more than one phase in those studies. Quanto [Gauderman and Morrison, 2006] was used for power calculations using the overall MAF of 0.43.

## Results

### Studies of Athletic Status

All studies described the types of sports or events performed in the athletic groups. All but one study stated that the HWE test was carried out and there was no evidence of departure among any of the control groups.

**Figure 1:**
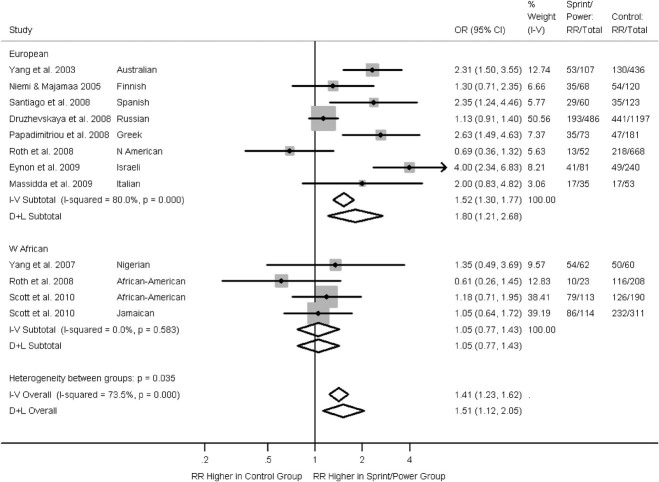
Associations between *ACTN3* R577X genotype (RR vs. RX + XX) and sprint/power athletic status from the literature. Arrow indicates the confidence interval extends beyond the plot axis. Stratified by ancestral group. Effects are given as odds ratios (OR) and 95% confidence intervals (CI). Points and the horizontal lines represent the study effect sizes and their 95% CIs. Sizes of the squares represent the weights of the studies. Diamonds represent the summary effects and their 95% CIs. I–V: inverse-variance, fixed effect model. D + L: DerSimonian & Laird, random effects model.

There were 10 studies comparing sprint/power athletes to controls, eight of which contained data on people described as Caucasians. The MAF in controls in seven of the eight studies in Europeans were between 0.39 and 0.49 (Supp. Table S1). The forest plot of the meta-analysis in Figure 1 shows that overall in Europeans there is evidence that the RR genotype is more common among sprint/power athletes than controls. However, there was significant heterogeneity (I^2^ = 80%, *P*<0.001), with one study reporting higher frequencies of RR among the controls, although its result was consistent with chance. The Egger test showed that there was little evidence to suggest the presence of “small study effects” for the eight studies on Europeans (*P* = 0.2; funnel plot presented in Supp. Fig. S2). Similar results were found after the removal of the initial study, the removal of the study in Europeans reporting a lower MAF of 0.32 (data not shown), and stratifying by sex (Supp. Fig. S3). There was no evidence for an association among non-Europeans.

**Figure 2:**
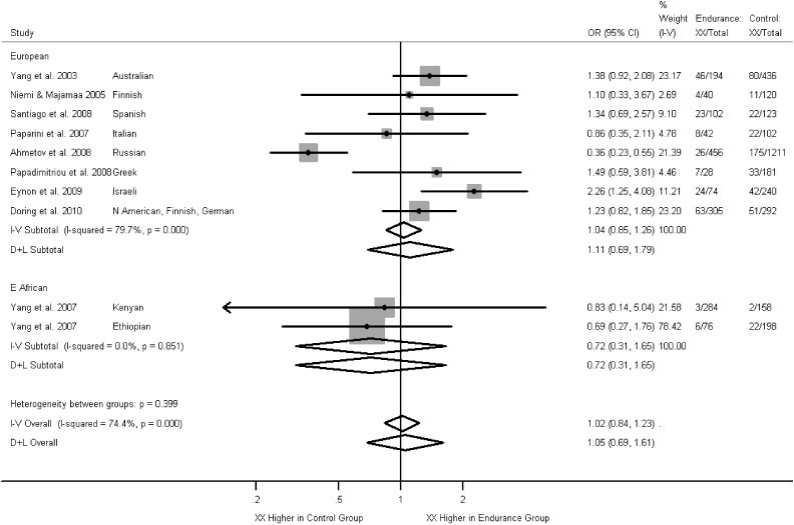
Associations between ACTN3 R577X genotype (XX vs. RX + RR) and endurance athletic status from the literature. Arrow indicates the confidence interval extends beyond the plot axis. Stratified by ancestral group. Effects are given as odds ratios (OR) and 95% confidence intervals (CI). Points and the horizontal lines represent the study effect sizes and their 95% CIs. Sizes of the squares represent the weights of the studies. Diamonds represent the summary effects and their 95% CIs. I–V: inverse-variance, fixed effect model. D + L: DerSimonian & Laird, random effects model.

There were nine studies included in the meta-analysis comparing endurance athletes with controls. Overall, there was no evidence that the X allele is more common among endurance athletes under the recessive model (Fig. 2), although there was significant heterogeneity in Europeans (I^2^ = 80%, *P*<0.001), with one study finding the XX genotype more common among controls. Similarly, there was no evidence under the dominant model (Supp. Fig. S4) or when considering males and females separately (Supp. Figs. S5 and S6).

Five studies included both sprint/power and endurance athletes. There was evidence that the RR genotype was more common among sprint/power athletes compared with endurance athletes (Supp. Fig. S7).

### Studies of Physical Capability

Relevant genotypic and phenotypic information were available for a total of 17,835 individuals aged between 10 and 90+ years old (Table 1 and Supp. Table S2). The quality of the genotyping was good with call rates exceeding 94% and the HWE condition being met (*P*>0.07). In ELSA, the study with the widest age range, the genotype frequencies were similar in those under and over 70 years in both the males and females (data not shown).

Supp. Table S3 shows there was no evidence for an association between R577X and height, weight, BMI, or WHR in either males or females (pooled *P*>0.1). As there was no evidence for associations with BMI, it was not included in the models for physical capability. No associations were found between level of physical activity and R577X (Supp. Table S4) in either sex.

**Figure 3:**
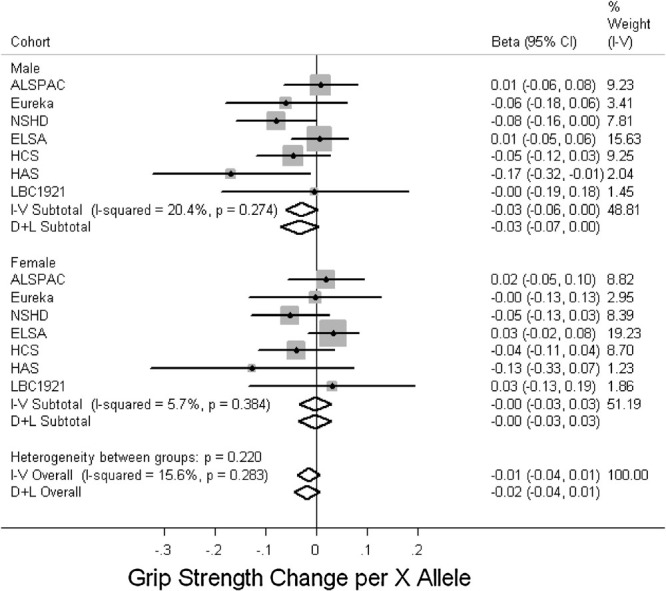
Associations between *ACTN3* R577X genotype and grip strength. Studies ordered by overall median age. Effects are given as per X allele change in grip strength (*z*-score) and 95% confidence intervals (CI). Points and the horizontal lines represent the study effect sizes and their 95% CIs. Sizes of the squares represent the weights of the studies. Diamonds represent the summary effects and their 95% CIs. I–V: inverse-variance, fixed effect model. D + L: DerSimonian & Laird, random effects model.

**Figure 4:**
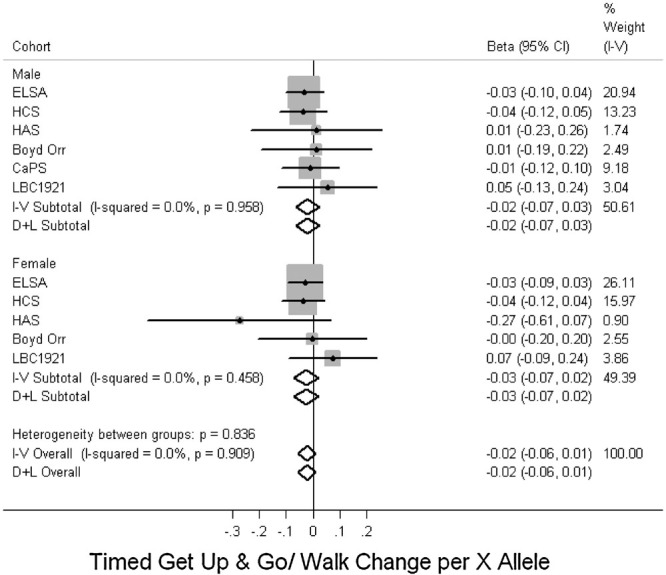
Associations between *ACTN3* R577X genotype and timed get up and go/walk. Studies ordered by overall median age. Effects are given as per X allele change in timed get up and go or walk (*z*-score) and 95% confidence intervals (CI). Points and the horizontal lines represent the study effect sizes and their 95% CIs. Sizes of the squares represent the weights of the studies. Diamonds represent the summary effects and their 95% CIs. I–V: inverse-variance, fixed effect model. D + L: DerSimonian & Laird, random effects model.

**Figure 5:**
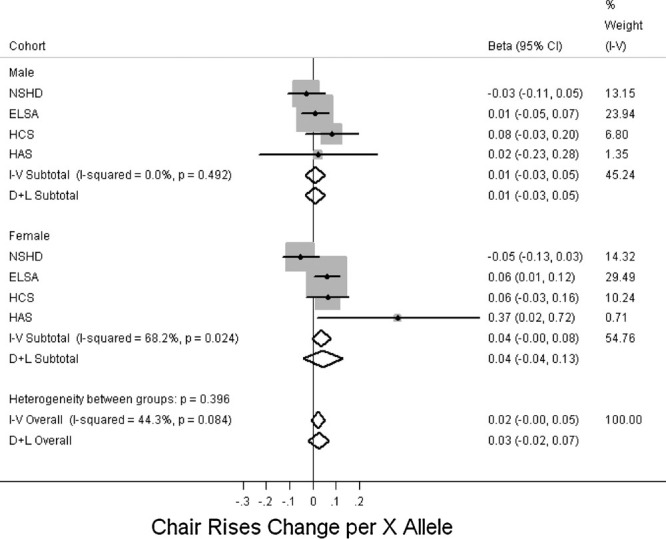
Associations between *ACNT3* R577X genotype and timed chair rises. Studies ordered by overall median age. Effects are given as per X allele change in timed chair rises (*z*-score) and 95% confidence intervals (CI). Points and the horizontal lines represent the study effect sizes and their 95% CIs. Sizes of the squares represent the weights of the studies. Diamonds represent the summary effects and their 95% CIs. I–V: inverse-variance, fixed effect model. D + L: DerSimonian & Laird, random effects model.

**Figure 6:**
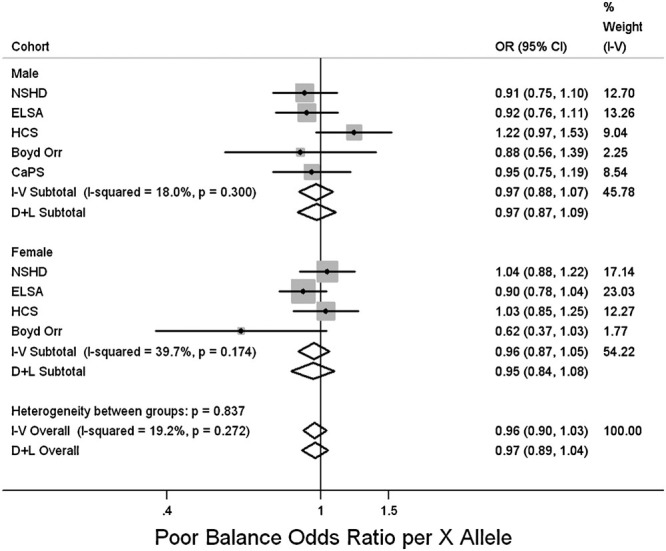
Associations between *ACTN3* R577X genotype and poor standing balance. Poor standing balance defined as inability to complete 30 sec, or 5 sec of the full tandem in ELSA. Studies ordered by overall median age. Effects are given as poor balance odds ratio (OR) per X allele and 95% confidence intervals (CI). Points and the horizontal lines represent the study effect sizes and their 95% CIs. Sizes of the squares represent the weights of the studies. Diamonds represent the summary effects and their 95% CIs. I–V: inverse-variance, fixed effect model. D + L: DerSimonian & Laird, random effects model.

Figure 3 and Supp. Table S5 show a trend toward an association between the R allele and better grip strength in males (*P* = 0.09) and although this was not seen in females, there was no evidence for a sex difference in this association (*P* = 0.2 for heterogeneity between males and females). The results were similar after adjusting for height. In addition, there was no evidence of associations with grip strength in the follow-up phases adjusting for its measure at a previous phase in HCS, HAS, or LBC1921 (data not shown). There was no evidence for an association with other measures of physical capability (Figs. 4–6, Supp. Table S5).

After stratifying by physical activity in the analyses of anthropometric and physical capability traits, results did not differ substantially, except for chair rises in females, where a significant association in ELSA was only seen in the physically active group (*P*-value for interaction in ELSA = 0.008; data not shown).

The forest plots, ordered by median study age, showed no consistent trend in effect sizes for the studies.

In only a small number of tests did the full genotype model represent a significantly better fit than the per allele model: physical activity and timed walk in LBC1921 females and standing balance in NSHD males.

## Discussion

We conducted a meta-analysis of results from the published literature on the associations between *ACTN3* R577X and athletic status, as well as investigated associations between this polymorphism and physical capability and anthropometry phenotypes in 17,835 preadolescent, adolescent, and older individuals from nine studies of the general population. We found evidence that among Europeans the RR genotype is more common among sprint/power athletes compared with their controls, but there was little evidence to support an association with *ACTN3* R577X and physical capability.

The initial report [Yang et al., 2003] of an overrepresentation of the R allele in sprint/power athletes compared with controls also hypothesized that the XX genotype may be advantageous to endurance athleticism. Since then, a number of findings have replicated the association with sprint/power performance [Druzhevskaya et al., 2008; Eynon et al., 2009b; Moran et al., 2007; Niemi and Majamaa, 2005; Papadimitriou et al., 2008; Roth et al., 2008; Santiago et al., 2008], but there has been little evidence to support the role of the XX genotype in improving endurance athleticism. One study in Europeans [Eynon et al., 2009b] found an overrepresentation of the XX genotype in endurance athletes, but conversely, another investigation found a lower XX genotype frequency among endurance athletes [Ahmetov et al., 2008]. The meta-analysis reported here shows that, although there was significant heterogeneity, based on the current literature there is evidence to support the hypothesis that the RR genotype is more common among sprint/power athletes compared with controls in both male and female Europeans. Although there were too few studies to formally investigate the heterogeneity with meta-regression (Cochrane Handbook for Systematic Reviews of Interventions, Version 5.0.2), there appeared to be no relationship between the study effect sizes and the proportion of athletes at international level. We found no evidence for “small study effects” among the studies in Europeans, although the test is likely to be underpowered here given the small number of published studies [Sterne et al., 2000]. Although sprint/power athletes differed from endurance athletes, we found no evidence that endurance athletes differed from controls with respect to the R577X genotype; therefore, the findings can be summarized as a sprint/power versus nonsprint/nonpower athlete association.

Type IIb muscle fibers are fast twitch white fibers fueled by glycolysis and glycogen, which contrast with type IIa fast twitch fibers and type I slow twitch fibers, both of which are red (myoglobin rich) and generate ATP by oxidative phosphorylation. Type IIb fibers fatigue easily as their energy supply cannot be sustained by glycolysis and glycogen. An earlier report [Vincent et al., 2007] indicated a difference of type IIb muscle fiber content, 9 versus 14% of total fibers in *vastus lateralis* in XX versus RR (*P* = 0.04), and also showed a major difference, as expected, of *ACTN3* content according to genotype. A larger study [Norman et al., 2009] did not find a fiber content difference, or effects on power output, fatigability or force–velocity relationship, but did find that repeated exercise bouts may increase dynamic torque generation in RR genotype by 4–10%, dependent on angular velocity. *ACTN2* upregulation may partially compensate *ACTN3* activity differences [Norman et al., 2009]. In a mouse *ACTN3* knockout, skeletal muscle showed increased glycogen by 5–30% according to which muscle, lower glycogen phosphorylase, which is rate limiting in glycogen usage, and altered calcium signaling [Quinlan et al., 2010]. Thus, although the effects at the gene expression level are qualitative, at the structural and metabolic levels they are more subtle, although apparently translating also into an overrepresentation of RR genotype in sprint/power athletes (Fig. 1).

From our population-based studies we found no evidence for an association between R577X and the anthropometric or physical capability measures, even before correcting for multiple testing. There was, however, a trend toward 577R male carriers having better grip strength, a power-orientated phenotype, although this was consistent with chance (*P* = 0.09). The lack of association found with our physical capability traits suggests that although the *ACTN3* R577X SNP may be associated with sprint/power athletic status, it contributes little or not at all to the interindividual variability in the capacity to undertake the physical tasks of daily living in the general population at any stage of life.

From the previous smaller studies carried out in the general population, the X allele has been associated with lower knee extensor peak torque in women (although not men) [Walsh et al., 2008] and men [Vincent et al., 2007], lower midthigh cross-sectional area in women [Zempo et al., 2010], lower elbow maximal voluntary contraction strength in women (not men) [Clarkson et al., 2005], greater decline in 5-year 400-m walk speeds in men (not women) [Delmonico et al., 2008], and increased risk in women of persistent lower extremity limitation (not men) [Delmonico et al., 2008] and falls [Judson et al., 2010]. In our study of physical capability, sample size calculations for the quantitative traits estimated that around 3,300 individuals would be required to detect a beta coefficient of 0.07 *z*-score units, or around 6,400 individuals for 0.05 *z*-score units, with 80% power at the 5% significance level. In grip strength, for example, 0.05 *z*-score units would correspond to a difference between the RR and XX groups of around 0.8 kg in men and 0.6 kg in women, assuming standard deviations of 8 and 6, respectively. Such effects on physical capability in older age would be of interest and are also potentially relevant to the loss of muscle mass with age due to muscle atrophy. They may also be relevant to the mechanisms of response to therapeutic strategies such as functional electrical stimulation used in rehabilitation [Squecco et al., 2009] and to strategies that could, for example, alter muscle glycogen content. We had sufficient power to detect differences as small as 0.07 *z*-score units for all our traits in males and females; therefore, if there are any associations between *ACTN3* R577X and these physical capability traits in adolescents or older adults, they are very modest. Indeed, one other study of older adults found no associations between R577X and 6-m walk times, grip strength, and five chair rise times [Delmonico et al., 2008]. However, it remains possible that *ACTN3* genotype is of consequence when considered in conjunction with other genetic variants, and it is likely to be only one of many polymorphisms that compose a genetic profile that is beneficial to sprint/power performance [Ruiz et al., 2010]. Also, much work remains to be done on the mode of action of *ACTN3* genotype on sprint/power performance.

### Limitations

Although our aim was to include all publications investigating differences in genotype frequencies in athletes and nonathletes, we were unable to get frequencies from one large study [Saunders et al., 2007] on Ironman triathletes, although the lack of association found in that study is consistent with the results from our meta-analysis on endurance athletic status using all other studies. The absence of precise athletic phenotypes is clear in this meta-analysis, as the composition of the endurance and sprint/power athletic groups is diverse, with different sports and levels of achievement among the athletes. The majority of the studies sampled controls from the general population and although the current low number of studies limits formal investigation into the effects of using sedentary sex-matched controls instead, it is suggested that future investigations should take information such as the physical activity levels measured objectively from the use of accelerometers, for example, of their controls into consideration either with appropriate subgroup analysis or in the selection of the controls. In populations of primarily African ancestry, there was no apparent effect of R577X genotype on sprint/power athletic status. However, statistical power may be low due to the small number of studies and the lower X allele frequency in African populations [MacArthur et al., 2007; Yang et al., 2007]. It is also possible that environmental factors or other genetic factors might modify the R577X effects. Investigations in other non-European populations are also warranted.

In our studies of physical capability the levels of physical activity were derived from self reports. Although we found no association between R577X and physical activity, as expected given both athletic groups would be considered highly physically active, the lack of precise objective measures may have limited our ability to identify subtle interaction effects between physical activity levels and R577X on physical capability. The lack of evidence found for associations with physical capability traits from our nine studies do not rule out an association of small magnitude between R577X and the other phenotypes reported in smaller population-based studies. Rather, studies with greater statistical power are required to satisfactorily investigate those traits as well as resolve the observed sex differences found for them.

## Conclusion

Our meta-analysis of results from the published literature provides evidence for an overrepresentation of the *ACTN3* R577X RR genotype in sprint/power athletes in Europeans, although it does not support the hypothesis that the X allele is advantageous to endurance athletic status. We found little evidence of associations between R577X and physical capability traits in adolescence or later in the life course.
