# Supplementary material for: ACTN3 Genotype, Athletic Status, and Life Course Physical Capability: Meta-Analysis of the Published Literature and Findings from Nine Studies
Source: Hum Mutat. 2011 May 3;32(9):1008–18. doi: 10.1002/humu.21526 (PMC3174315; doi:10.1002/humu.21526)
Supplement: Supplementary file 1 [file humu0032-1008-SD1.pdf]

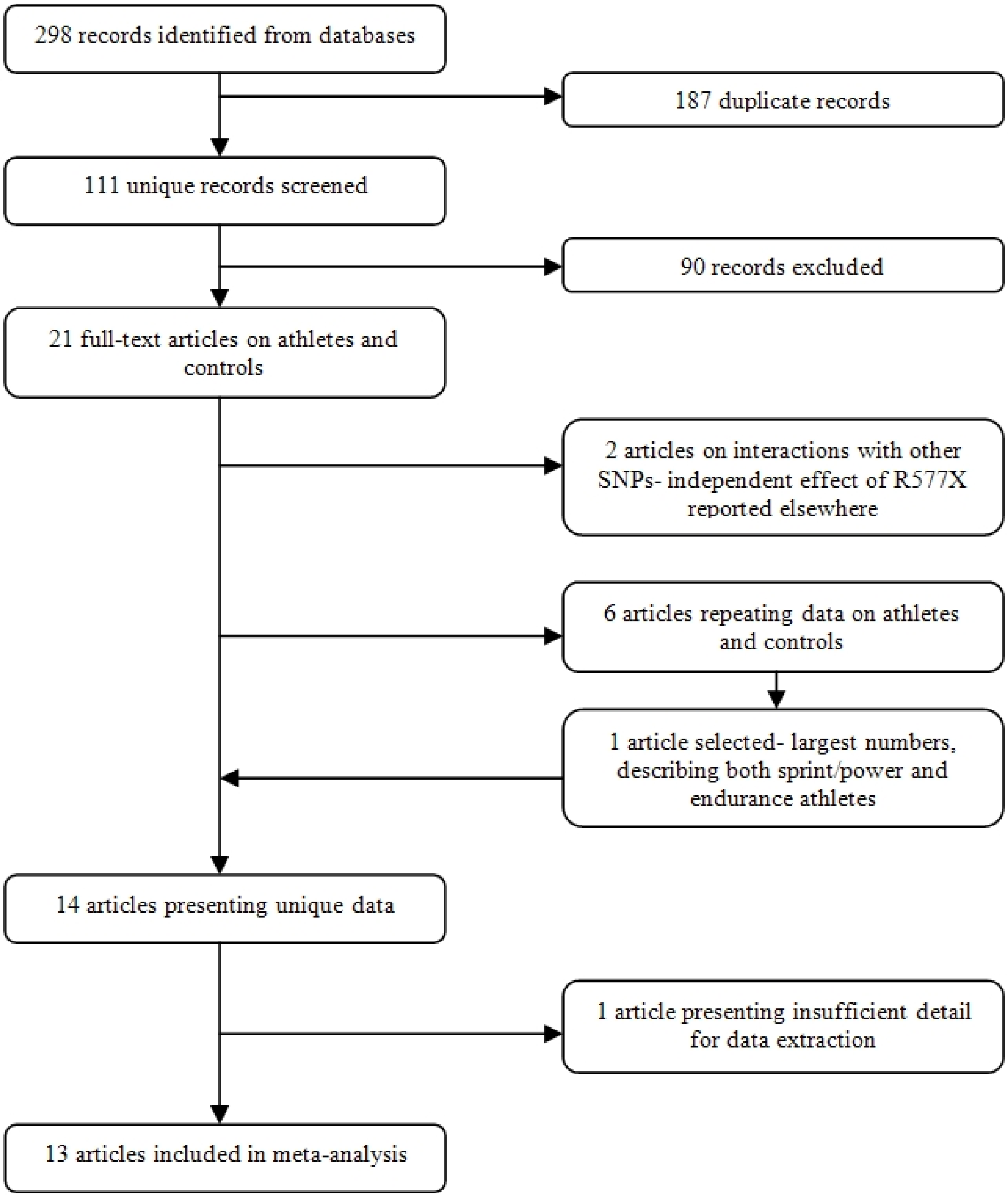

Supp. Figure S1. Flow of information for the identification of studies.

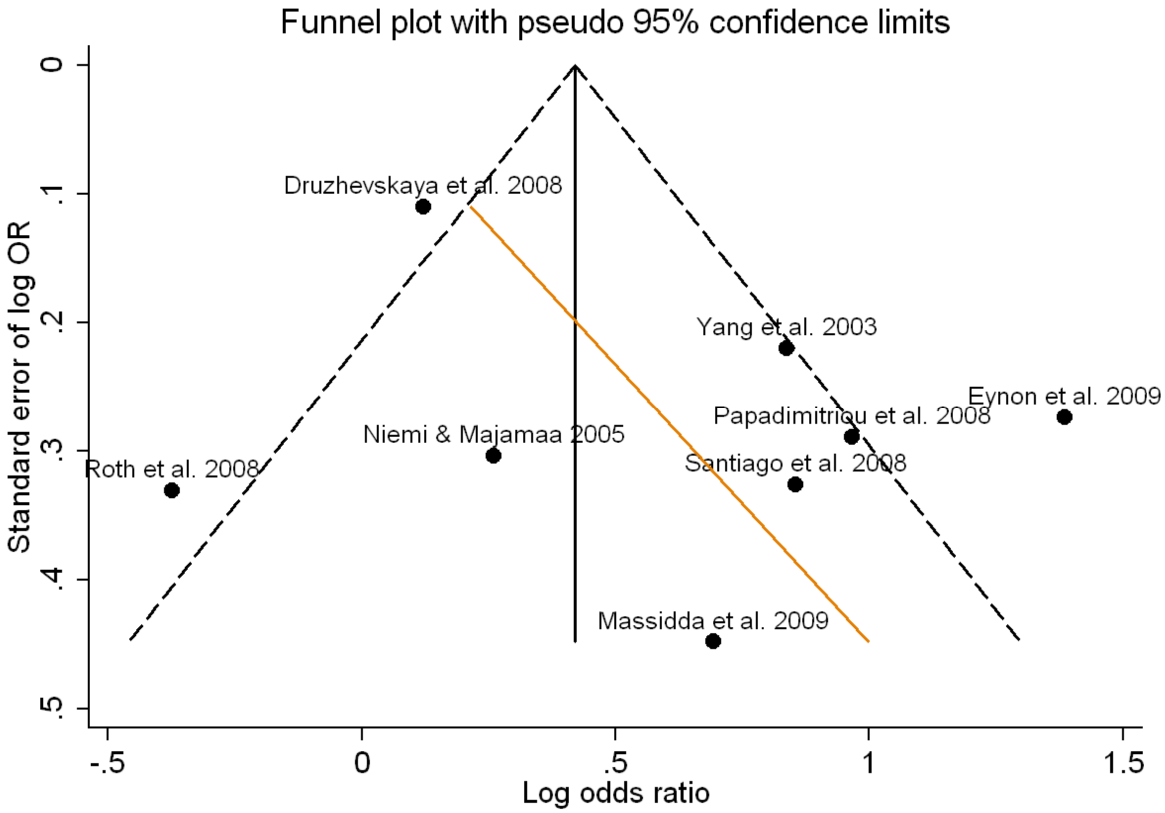

Supp. Figure S2. Funnel plot on eight studies for ACTN3 R577X Genotype (RR vs. RX+XX) and Sprint/Power Athletic Status in Europeans.Vertical line represents the overall estimate. Coloured slope represents the fitted regression line for funnel plot asymmetry. Dashed lines represent the pseudo 95% confidence limits. OR: odds ratio.

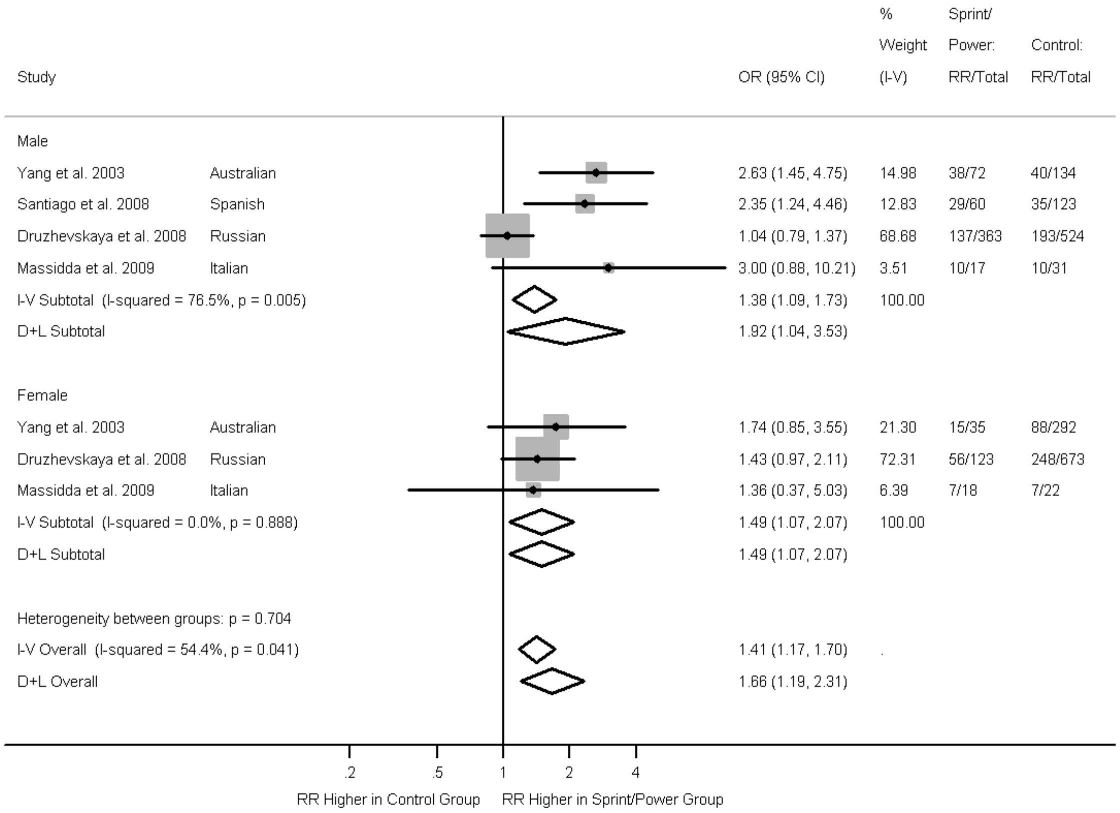

Supp. Figure S3. Associations between ACTN3 R577X Genotype (RR vs. RX+XX) and Sprint/Power Athletic Status by Sex from the Literature. Effects are given as odds ratios (OR) and 95% confidence intervals (CI). Analysis limited to studies with data presented by sex. Points and the horizontal lines represent the study effect sizes and their 95% CIs. Sizes of the squares represent the weights of the studies. Diamonds represent the summary effects and their 95% CIs. I-V: Inverse-variance, fixed effect model. D+L: DerSimonian & Laird, random effects model.

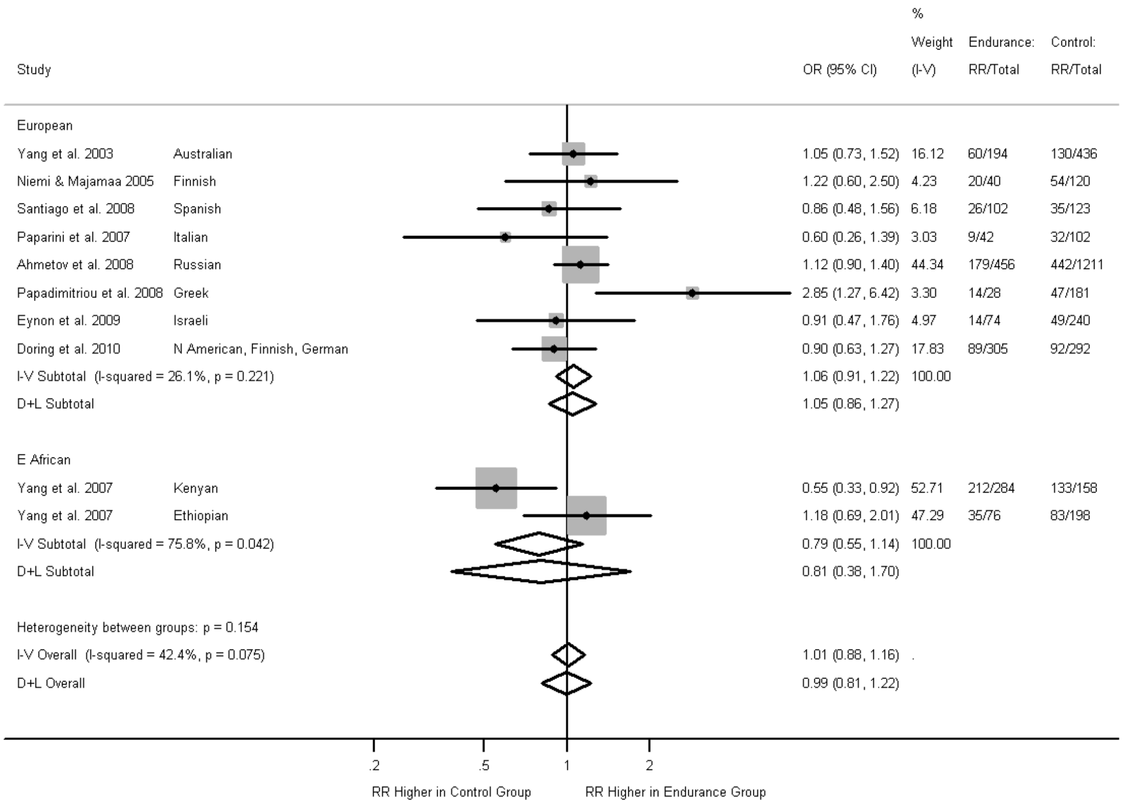

Supp. Figure S4. Associations between ACTN3 R577X Genotype (RR vs. RX+XX) and Endurance Athletic Status from the Literature. Arrow indicates the confidence interval extends beyond the plot axis. Stratified by ancestral group. Effects are given as odds ratios (OR) and 95% confidence intervals (CI). Points and the horizontal lines represent the study effect sizes and their 95% CIs. Sizes of the squares represent the weights of the studies. Diamonds represent the summary effects and their 95% CIs. I-V: Inverse-variance, fixed effect model. D+L: DerSimonian & Laird, random effects model.

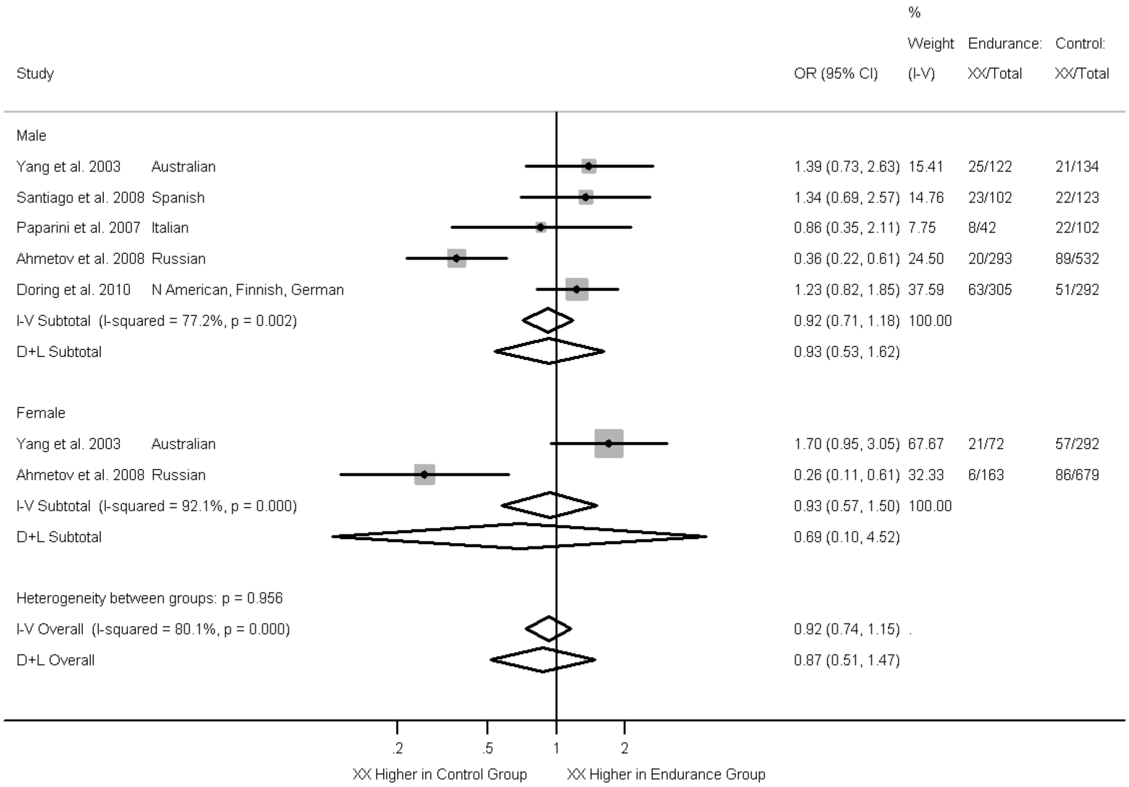

Supp. Figure S5. Associations between ACTN3 R577X Genotype (XX vs. RX+RR) and Endurance Athletic Status by Sex from the Literature. Effects are given as odds ratios (OR) and 95% confidence intervals (CI). Analysis limited to studies with data presented by sex. Points and the horizontal lines represent the study effect sizes and their 95% CIs. Sizes of the squares represent the weights of the studies. Diamonds represent the summary effects and their 95% CIs. I-V: Inverse-variance, fixed effect model. D+L: DerSimonian & Laird, random effects model.

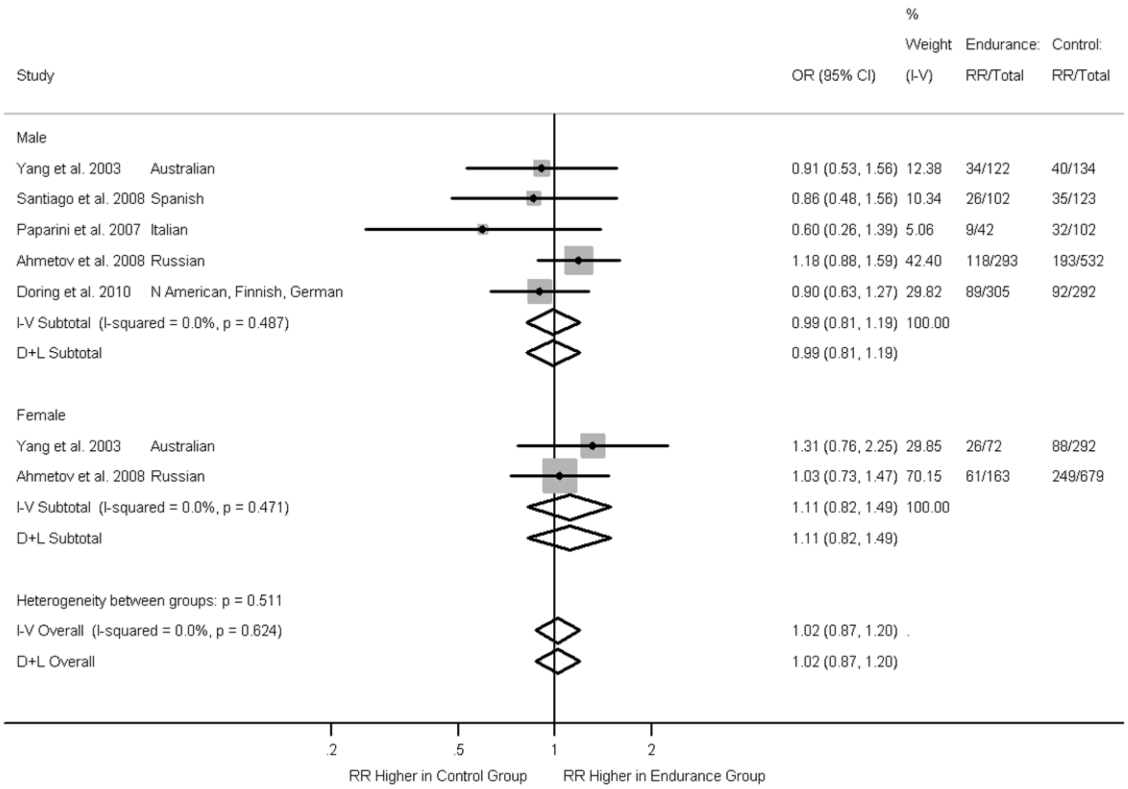

Supp. Figure S6. Associations between ACTN3 R577X Genotype (RR vs. RX+XX) and Endurance Athletic Status by Sex from the Literature. Effects are given as odds ratios (OR) and 95% confidence intervals (CI). Analysis limited to studies with data presented by sex. Points and the horizontal lines represent the study effect sizes and their 95% CIs. Sizes of the squares represent the weights of the studies. Diamonds represent the summary effects and their 95% CIs. I-V: Inverse-variance, fixed effect model. D+L: DerSimonian & Laird, random effects model.

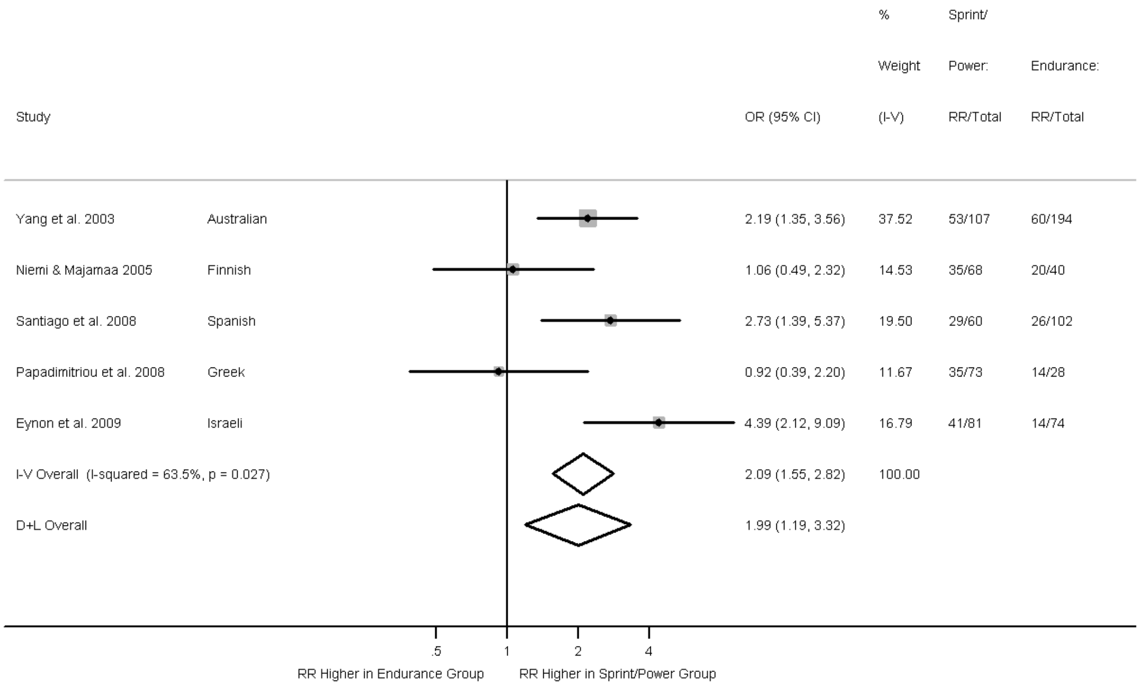

Supp. Figure S7. Associations between ACTN3 R577X Genotype (RR vs. RX+XX) and Sprint/Power and Endurance Athletic Status from the Literature. Effects are given as odds ratios (OR) and 95% confidence intervals (CI). Analysis limited to studies with data on both sprint/power and endurance athletes. Points and the horizontal lines represent the study effect sizes and their 95% CIs. Sizes of the squares represent the weights of the studies. Diamonds represent the summary effects and their 95% CIs. I-V: Inverse-variance, fixed effect model. D+L: DerSimonian & Laird, random effects model.

Supp. Table S1. Summary of the Phenotypic Categorisations used in the Meta-analysis from the Published Literature on *ACTN3* R577X Genotype and Athletic Status

Study	Population	Sprint/Power Sport or Event	Endurance Sport or Event	Source of Controls	X Allele Frequency in Controls
Yang *et al.* 2003	Australian	30% competed at Olympic level: Track athletes (800m or less), swimmers (200m or less), judo athletes, short-distance track cyclists, and speed skaters.	9% competed at Olympic level: Long-distance cyclists, rowers, swimmers (400m or more), track athletes (5,000m or more), and cross-country skiers.	Blood donors, children and adults.	0.44
Niemi & Majamaa 2005	Finnish	34% competed at European or World Championship level: Sprinters (100 to 400m) and field athletes.	50% competed at European or World Championship level: Runners (800m to marathon) and walkers.	Blood donors.	0.32
Yang *et al.* 2007	Ethiopian		Ethiopian athletics squad members (3000m to marathon).	Population.	0.35
	Kenyan		24% competed at international level: Distance runners (3000m to marathon).	Population.	0.09
	Nigerian	32% competed at international level: Track runners (400m or less) and jump athletes (110m hurdles, long and triple jumps).		Population.	0.08
Paparini *et al.* 2007	Italian		Rowers in pre-Olympic training.	Population.	0.45
Papadimitriou *et al.* 2008	Greek	60% competed at Olympic or European level: Track athletes (100 to 400m), jumpers, throwers and decathletes.	50% competed at Olympic or European level: Track athletes (800m to marathon), triathletes and walkers.	Population.	0.46
Roth *et al.* 2008	N American (White)	24% ranked in world top 100: Body builders, power lifters and strength athletes.		Population.	0.44
	African-American				0.25
Druzhevskaya *et al.* 2008	Russian	63% competed at international level (including Olympic): Alpine skiers, artistic gymnasts, body builders, figure skaters, ice hockey players, jumpers, power lifters, runners (100 to 400m), ski jumpers, soccer players, speed skaters, swimmers (50 to 100m), throwers, volleyball players,		Population.	0.39

**Study**	**Population**	**Sprint/Power Sport or Event**	**Endurance Sport or Event**	**Source of Controls**	**X Allele Frequency in Controls**
		weight lifters and wrestlers.			
Ahmetov *et al.* 2008	Russian		51% competed at international level (including Olympic): biathletes (15 to 20km), cross-country skiers (10 to 50km), race walkers (10 to 50km), road cyclists (more than 50km), rowers (more than 2000m), swimmers (0.8 to 25km) and triathletes (swimming 1500m, cycling 40km, running 10km).	Population.	0.39
Santiago *et al.* 2008	Spanish	22% competed at international level: Soccer players.	Cyclists (Tour de France finishers) and Olympic level runners (1500m to marathon).	Sedentary adults.	0.45
Massidda *et al.* 2009	Italian	International and Olympic artistic gymnasts.		Sedentary.	0.43
Eynon *et al.* 2009	Israeli	32% competed at international level (including Olympic): Sprinters (100 to 200m).	27% competed at international level (including Olympic): Runners (10km, marathon).	Sedentary population.	0.49
Doring *et al.* 2010	N American (Caucasian), Finnish, German		Competed at national or international level: Cross-country skiers, bi- and triathletes, cyclists, runners, and rowers.	Sedentary.	0.43
Scott *et al.* 2010	Jamaican	74% competed at international level: Sprinters (up to 400m), jumpers and throwers.		Population.	0.14
	African-American	96% competed at international level: Sprinters (up to 400m), jumpers and throwers.		Population.	0.19

Sports and levels summarised from publications.

References

Ahmetov II, Druzhevskaya AM, Astratenkova IV, Popov DV, Vinogradova OL, Rogozkin VA. 2008. The ACTN3 R577X polymorphism in Russian endurance athletes. Br J Sports Med 44:649-652.

Doring F, Onur S, Geisen U, Boulay M, Perusse L, Rankinen T, Rauramaa R, Wolfahrt B, Bouchard C. 2010. ACTN3 R577X and other polymorphisms are not associated with elite endurance athlete status in the Genathlete study. J of Sports Sc 28:1355-1359.

Druzhevskaya AM, Ahmetov II, Astratenkova IV, Rogozkin VA. 2008. Association of the ACTN3 R577X polymorphism with power athlete status in Russians. Eur J Appl Physiol 103:631-634.

Eynon N, Duarte JA, Oliveira J, Sagiv M, Yamin C, Meckel Y, Sagiv M, Goldhammer E. 2009. ACTN3 R577X polymorphism and Israeli top-level athletes. Int J Sports Med 30:695-698.

Massidda M, Vona G, Calò CM. 2009. Association between the ACTN3 R577X polymorphism and artistic gymnastic performance in Italy. Genet Test Mol Biomarkers 13:377-380.

Niemi A, Majamaa K. 2005. Mitochondrial DNA and ACTN3 genotypes in Finnish elite endurance and sprint athletes. Eur J Hum Genet 13:965-969.

Papadimitriou ID, Papadopoulos C, Kouvatsi A, Triantaphyllidis C. 2008. The ACTN3 gene in elite Greek track and field athletes. Int J Sports Med 29:352-355.

Paparini A, Ripani M, Giordano GD, Santoni D, Pigozzi F, Romano-Spica V. 2007. ACTN3 genotyping by real-time PCR in the Italian population and athletes. Med Sci Sports Exerc 39:810-815.

Roth SM, Walsh S, Liu D, Metter EJ, Ferrucci L, Hurley BF. 2008. The ACTN3 R577X nonsense allele is under-represented in elite-level strength athletes. Eur J Hum Genet 16:391-394.

Santiago C, González-Freire M, Serratosa L, Morate FJ, Meyer T, Gómez-Gallego F, Lucia A. 2008. ACTN3 genotype in professional soccer players. Br J Sports Med 42:71-73.

Scott RA, Irving R, Irwin L, Morrison E, Charlton V, Austin K, Tladi D, Deason M, Headley SA, Kolkhorst FW, Yang N, North K, Pitsiladis YP. 2010. ACTN3 and ACE Genotypes in Elite Jamaican and US Sprinters. Med Sci Sports Exerc 42:107-112.

Yang N, MacArthur DG, Gulbin JP, Hahn AG, Beggs AH, Easteal S, North K. 2003. ACTN3 Genotype Is Associated with Human Elite Athletic Performance. Am J Hum Genet 73:627-631.

Yang N, MacArthur DG, Wolde B, Onywera VO, Boit MK, Lau SYM, Wilson RH, Scott RA, Pitsiladis YP, North K. 2007. The ACTN3 R577X polymorphism in East and West African athletes. Med Sci Sports Exerc 39:1985-1988.

Supp. Table S2. Summary of Sex, Age and *ACTN3* R577X Genotype Frequencies by Cohort and Sex

Cohort	Age* in years, median (range)	Males			Females			Total Individuals
		RR n (%)	RX n (%)	XX n (%)	RR n (%)	RX n (%)	XX n (%)	
ALSPAC	11 (10-13)	487 (32.1)	722 (47.6)	308 (20.3)	467 (32.2)	706 (48.7)	277 (19.1)	2967
Eureka	15 (11-18)	176 (33.5)	250 (47.6)	99 (18.9)	159 (34.0)	225 (48.2)	83 (17.8)	992
NSHD	53	401 (31.1)	647 (50.2)	242 (18.8)	429 (32.9)	610 (46.7)	266 (20.4)	2595
ELSA	65 (52-90+)	813 (32.8)	1208 (48.8)	456 (18.4)	946 (32.0)	1415 (47.8)	597 (20.2)	5435
HCS	66 (59-73)	486 (32.6)	743 (49.8)	264 (17.7)	448 (33.5)	636 (47.5)	254 (19.0)	2831
HAS	67 (63-73)	100 (32.4)	148 (47.9)	61 (19.7)	74 (37.2)	94 (47.2)	31 (15.6)	508
Boyd Orr	70 (64-82)	87 (27.4)	163 (51.4)	67 (21.1)	103 (28.1)	199 (54.2)	65 (17.7)	684
CaPS	73 (65-83)	444 (33.9)	643 (49.1)	222 (17.0)	-	-	-	1309
LBC1921	79 (77-80)	65 (30.7)	98 (46.2)	49 (23.1)	93 (30.8)	152 (50.3)	57 (18.9)	514
Total	60 (10-90+)	3059 (32.4)	4622 (48.9)	1768 (18.7)	2719 (32.4)	4037 (48.1)	1630 (19.4)	17835

*Age at phase from which the majority of variables are taken, i.e. ALSPAC: Focus @11; Boyd Orr: III; CaPS: V; ELSA: II; HAS: I; HCS: I; LBC1921: I; NSHD: 1999 Collection. ALSPAC: Avon Longitudinal Study of Parents and Children; CaPS: Caerphilly Prospective Study; ELSA: English Longitudinal Study of Ageing; HAS: Hertfordshire Ageing Study; HCS: Hertfordshire Cohort Study; NSHD: National Survey of Health and Development.

Supp. Table S3. Anthropometry by *ACTN3* R577X Genotype and Sex

		Males					Females				
**Variable**	**Cohort**	**RR mean (sd) [n]**	**RX mean (sd) [n]**	**XX mean (sd) [n]**	**Total mean (sd) [n]**	**p-value**	**RR mean (sd) [n]**	**RX mean (sd) [n]**	**XX mean (sd) [n]**	**Total mean (sd) [n]**	**p-value**
Height, cm	ALSPAC	150.2 (7.0) [484]	150.2 (7.1) [714]	149.5 (6.6) [308]	150.1 (7.0) [1506]	0.16	151.1 (7.3) [462]	151.5 (7.2) [702]	151.5 (6.8) [276]	151.4 (7.2) [1440]	0.38
	Eureka	167.3 (10.6) [176]	167.8 (11.4) [250]	167.6 (10.8) [99]	167.6 (11.0) [525]	0.79	160.2 (7.1) [159]	159.9 (6.5) [225]	161.6 (5.6) [83]	160.3 (6.6) [467]	0.23
	NSHD	174.7 (6.4) [400]	174.6 (6.5) [647]	174.5 (6.7) [240]	174.6 (6.5) [1287]	0.69	162.1 (5.6) [429]	161.7 (5.9) [606]	161.6 (6.3) [265]	161.8 (5.9) [1300]	0.23
	ELSA	172.5 (7.0) [783]	173.3 (6.9) [1178]	173.0 (6.5) [439]	173.0 (6.8) [2400]	0.11	159.1 (6.5) [924]	159.8 (6.6) [1355]	159.8 (6.8) [582]	159.6 (6.6) [2861]	0.0211
	HCS	174.5 (6.4) [486]	174.0 (6.5) [741]	174.1 (6.5) [264]	174.2 (6.4) [1491]	0.23	160.7 (5.7) [448]	161.0 (6.0) [635]	160.9 (6.0) [254]	160.9 (5.9) [1337]	0.61
	HAS	172.9 (6.0) [100]	171.3 (6.8) [148]	171.6 (6.3) [61]	171.9 (6.5) [309]	0.14	158.8 (5.6) [74]	158.8 (6.3) [94]	159.3 (6.1) [31]	158.9 (6.0) [199]	0.77
	Boyd Orr	171.2 (7.0) [87]	171.5 (6.7) [163]	172.7 (6.1) [67]	171.7 (6.6) [317]	0.18	157.6 (7.7) [103]	158.7 (6.0) [199]	158.0 (4.9) [65]	158.3 (6.4) [367]	0.52
	CaPS	170.0 (6.1) [267]	170.3 (6.4) [402]	169.8 (5.9) [123]	170.1 (6.2) [792]	0.96	-	-	-	-	-
	LBC1921	170.5 (7.3) [65]	172.2 (6.5) [98]	171.4 (7.0) [49]	171.5 (6.9) [212]	0.45	158.1 (5.4) [93]	157.1 (6.1) [152]	157.2 (5.4) [56]	157.4 (5.8) [301]	0.31
	**Pooled**	**[2848]**	**[4341]**	**[1650]**	**[8839]**	**0.79**	**[2692]**	**[3968]**	**[1612]**	**[8272]**	**0.14**
Weight, kg	ALSPAC	43.3 (9.3) [455]	43.2 (9.3) [668]	41.9 (8.6) [283]	43.0 (9.2) [1406]	0.0498	43.8 (10.0) [466]	44.2 (9.8) [700]	44.8 (10.5) [277]	44.2 (10.0) [1443]	0.19
	Eureka	62.6 (15.1) [176]	63.5 (16.7) [250]	62.2 (13.8) [99]	63.0 (15.6) [525]	0.93	55.7 (10.2) [158]	56.5 (9.9) [225]	56.9 (9.5) [83]	56.3 (9.9) [466]	0.36
	NSHD	83.2 (12.4) [400]	83.5 (13.6) [646]	84.2 (13.3) [240]	83.5 (13.2) [1286]	0.38	72.1 (15.0) [425]	71.6 (13.9) [605]	70.2 (12.2) [264]	71.5 (14.0) [1294]	0.09
	ELSA	82.7 (14.8) [789]	83.2 (13.1) [1178]	83.4 (14.2) [447]	83.1 (13.8) [2414]	0.35	70.9 (14.2) [917]	71.0 (14.4) [1364]	70.6 (13.8) [582]	70.9 (14.2) [2863]	0.79
	HCS	83.1 (13.1) [486]	82.5 (12.8) [741]	81.7 (11.9) [264]	82.6 (12.7) [1491]	0.14	71.2 (13.1) [448]	71.3 (13.4) [636]	72.1 (14.3) [254]	71.4 (13.5) [1338]	0.45
	HAS	80.1 (11.3) [100]	78.5 (13.3) [148]	78.6 (11.3) [61]	79.1 (12.3) [309]	0.39	67.8 (11.0) [74]	69.0 (12.7) [94]	68.0 (14.1) [31]	68.4 (12.3) [199]	0.80
	Boyd Orr	79.9 (11.4) [86]	79.4 (13.0) [163]	81.1 (11.6) [67]	79.9 (12.3) [316]	0.58	68.2 (13.1) [103]	69.4 (13.2) [199]	70.9 (11.6) [65]	69.3 (12.9) [367]	0.19
	CaPS	80.2 (12.9) [268]	80.8 (12.5) [405]	82.2 (14.1) [124]	80.8 (12.9) [797]	0.18	-	-	-	-	-
	LBC1921	76.0 (11.3) [65]	77.3 (10.4) [98]	79.0 (11.7) [49]	77.3 (11.0) [212]	0.16	63.8 (9.8) [93]	65.5 (11.7) [152]	63.8 (12.3) [57]	64.6 (11.3) [302]	0.80
	**Pooled**	**[2825]**	**[4297]**	**[1634]**	**[8756]**	**0.94**	**[2684]**	**[3975]**	**[1613]**	**[8272]**	**0.55**
BMI, kg/m^2	ALSPAC	19.0 (3.1) [452]	18.9 (3.1) [661]	18.6 (2.9) [283]	18.9 (3.1) [1396]	0.06	19.0 (3.4) [461]	19.1 (3.3) [697]	19.3 (3.5) [276]	19.1 (3.4) [1434]	0.25
	Eureka	22.1 (3.8) [176]	22.3 (4.2) [250]	22.0 (3.6) [99]	22.2 (3.9) [525]	0.86	21.6 (3.4) [158]	22.0 (3.3) [225]	21.7 (3.0) [83]	21.9 (3.3) [466]	0.64
	NSHD	27.3 (3.9) [400]	27.3 (3.9) [646]	27.6 (4.0) [240]	27.4 (3.9) [1286]	0.28	27.4 (5.5) [425]	27.4 (5.4) [603]	27.0 (4.8) [263]	27.3 (5.3) [1291]	0.29
	ELSA	27.7 (4.3) [775]	27.7 (3.9) [1162]	27.9 (4.2) [434]	27.7 (4.1) [2371]	0.60	28.0 (5.3) [906]	27.9 (5.3) [1336]	27.6 (4.9) [576]	27.9 (5.2) [2818]	0.15
	HCS	27.3 (3.9) [486]	27.2 (3.8) [740]	27.0 (3.5) [264]	27.2 (3.8) [1490]	0.35	27.6 (4.9) [448]	27.5 (4.8) [635]	27.8 (5.2) [254]	27.6 (4.9) [1337]	0.57
	HAS	26.8 (3.2) [100]	26.7 (3.7) [148]	26.8 (4.0) [61]	26.7 (3.6) [309]	0.96	26.9 (4.3) [74]	27.3 (4.6) [94]	26.7 (5.2) [31]	27.1 (4.6) [199]	0.93
	Boyd Orr	27.2 (3.4) [86]	27.0 (4.0) [163]	27.2 (3.4) [67]	27.1 (3.7) [316]	0.91	27.5 (5.3) [103]	27.5 (4.9) [199]	28.4 (4.4) [65]	27.7 (4.9) [367]	0.32
	CaPS	27.7 (4.0) [267]	27.8 (3.9) [402]	28.5 (4.7) [123]	27.9 (4.1) [792]	0.10	-	-	-	-	-
	LBC1921	26.1 (3.5) [65]	26.1 (3.5) [98]	26.9 (3.7) [49]	26.3 (3.5) [212]	0.29	25.5 (3.8) [93]	26.6 (4.6) [152]	25.9 (4.9) [56]	26.1 (4.5) [301]	0.40
	**Pooled**	**[2807]**	**[4270]**	**[1620]**	**[8697]**	**0.87**	**[2668]**	**[3941]**	**[1604]**	**[8213]**	**0.93**
Waist-hip ratio	ALSPAC	0.85 (0.05) [440]	0.85 (0.05) [663]	0.84 (0.05) [290]	0.85 (0.05) [1393]	0.10	0.82 (0.05) [423]	0.82 (0.05) [645]	0.82 (0.05) [255]	0.82 (0.05) [1323]	0.65
	NSHD	0.94 (0.06) [399]	0.93 (0.06) [647]	0.94 (0.07) [241]	0.94 (0.06) [1287]	0.83	0.80 (0.07) [427]	0.80 (0.06) [606]	0.81 (0.07) [265]	0.81 (0.07) [1298]	0.23
	ELSA	0.95 (0.06) [797]	0.95 (0.06) [1178]	0.96 (0.06) [446]	0.95 (0.06) [2421]	0.15	0.84 (0.06) [923]	0.84 (0.07) [1371]	0.84 (0.07) [585]	0.84 (0.06) [2879]	0.40
	HCS	0.97 (0.06) [486]	0.96 (0.06) [741]	0.96 (0.06) [263]	0.97 (0.06) [1490]	0.10	0.85 (0.07) [446]	0.85 (0.06) [633]	0.85 (0.06) [252]	0.85 (0.06) [1331]	0.32
	HAS	0.94 (0.06) [100]	0.93 (0.05) [147]	0.93 (0.04) [61]	0.93 (0.05) [308]	0.23	0.80 (0.05) [74]	0.80 (0.05) [94]	0.79 (0.05) [31]	0.80 (0.05) [199]	0.40
	Boyd Orr	0.98 (0.07) [50]	0.97 (0.09) [85]	0.97 (0.06) [43]	0.97 (0.07) [178]	0.67	0.86 (0.08) [56]	0.87 (0.08) [110]	0.86 (0.07) [40]	0.86 (0.08) [206]	0.90
	CaPS	0.93 (0.06) [442]	0.93 (0.06) [639]	0.94 (0.07) [221]	0.93 (0.06) [1302]	0.08	-	-	-	-	-
	**Pooled**	**[2714]**	**[4100]**	**[1565]**	**[8379]**	**0.73**	**[2349]**	**[3459]**	**[1428]**	**[7236]**	**0.70**

P-values per X allele. Pooled results based on z-scores. Waist-hip ratio in CaPS from Phase III.

Supp. Table S4. Physical Activity by *ACTN3* R577X Genotype and Sex

Description	Cohort	Males RR n (%)[a]	Males RX n (%)[a]	Males XX n (%)[a]	Males Total n (%)[a]	Males p-value	Females RR n (%)[a]	Females RX n (%)[a]	Females XX n (%)[a]	Females Total n (%)[a]	Females p-value
At least 2hrs/week	Eureka	132 (75.0)	191 (76.4)	73 (73.7)	396 (75.4)	0.90	93 (58.5)	116 (51.6)	45 (54.2)	254 (54.4)	0.38
At least once/month	NSHD	213 (53.1)	335 (51.9)	128 (53.1)	676 (52.5)	0.93	198 (46.2)	320 (52.5)	132 (49.6)	650 (49.8)	0.24
Vigorous at least once/month	ELSA	376 (46.2)	594 (49.2)	213 (46.7)	1183 (47.8)	0.67	313 (33.1)	523 (37.0)	212 (35.5)	1048 (35.4)	0.22
At least once/month	CaPS	133 (53.6)	203 (54.1)	63 (51.6)	399 (53.6)	0.79	-	-	-	-	-
At least once/month	LBC1921	44 (75.9)	49 (55.7)	23 (54.8)	116 (61.7)	0.0225	38 (47.5)	45 (36.3)	29 (58.0)	112 (44.1)	0.45
	**Pooled**	**[898/1696]**	**[1372/2567]**	**[500/960]**	**[2770/5223]**	**0.62**	**[642/1614]**	**[1004/2374]**	**[418/996]**	**[2064/4984]**	**0.15**

a: #participants with event (%); Pooled: [#participants with event/total # participants with relevant data]. P-values per X allele.

Supp. Table S5. Physical Capability by *ACTN3* R577X Genotype and Sex

Variable	Cohort	Males RR mean (sd) [n]	Males RX mean (sd) [n]	Males XX mean (sd) [n]	Males Total mean (sd) [n]	Males p-value	Females RR mean (sd) [n]	Females RX mean (sd) [n]	Females XX mean (sd) [n]	Females Total mean (sd) [n]	Females p-value
Grip strength, kg	ALSPAC	20.5 (4.1) [455]	20.8 (4.4) [687]	20.5 (4.1) [281]	20.7 (4.2) [1423]	0.81	19.6 (4.5) [446]	19.6 (4.2) [656]	19.8 (4.2) [262]	19.7 (4.3) [1364]	0.60
	Eureka	34.6 (10.3) [176]	34.4 (9.9) [250]	33.3 (9.1) [99]	34.3 (9.9) [525]	0.34	24.5 (5.0) [158]	24.6 (4.7) [225]	24.5 (4.7) [83]	24.5 (4.8) [466]	0.97
	NSHD	48.8 (11.8) [387]	47.4 (11.8) [625]	47.0 (13.4) [235]	47.8 (12.1) [1247]	0.05	28.2 (7.7) [413]	27.7 (8.1) [582]	27.5 (8.2) [256]	27.8 (8.0) [1251]	0.19
	ELSA	40.8 (10.0) [811]	41.3 (9.4) [1201]	40.7 (9.9) [452]	41.0 (9.7) [2464]	0.84	24.2 (6.7) [936]	24.6 (6.4) [1392]	24.6 (6.5) [593]	24.4 (6.5) [2921]	0.19
	HCS	44.5 (7.8) [486]	43.9 (7.1) [743]	43.9 (8.0) [263]	44.1 (7.5) [1492]	0.23	26.7 (5.7) [448]	26.6 (5.8) [636]	26.2 (5.7) [254]	26.6 (5.8) [1338]	0.32
	HAS	39.3 (6.6) [100]	37.7 (6.6) [148]	37.1 (7.8) [61]	38.1 (6.9) [309]	0.0361	22.9 (5.5) [73]	21.8 (5.3) [94]	21.8 (4.3) [31]	22.2 (5.2) [198]	0.22
	LBC1921	35.5 (6.7) [65]	33.9 (7.5) [98]	35.7 (8.2) [49]	34.8 (7.5) [212]	0.98	20.6 (3.9) [93]	20.6 (5.1) [152]	20.9 (4.0) [56]	20.7 (4.5) [301]	0.71
	**Pooled**	**[2480]**	**[3752]**	**[1440]**	**[7672]**	**0.09**	**[2567]**	**[3737]**	**[1535]**	**[7839]**	**0.90**
2.44m walk, s[a]	ELSA	0.99 (0.34) [515]	0.97 (0.35) [777]	0.98 (0.34) [292]	0.98 (0.34) [1584]	0.38	1.07 (0.39) [619]	1.05 (0.37) [867]	1.05 (0.36) [379]	1.06 (0.37) [1865]	0.36
3m get up and go, s[a]	HCS	2.35 (0.17) [331]	2.35 (0.17) [524]	2.34 (0.17) [181]	2.35 (0.17) [1036]	0.42	2.39 (0.21) [387]	2.38 (0.21) [528]	2.38 (0.18) [227]	2.38 (0.20) [1142]	0.37
3m get up and go, s[a]	HAS	2.48 (0.24) [51]	2.46 (0.21) [55]	2.50 (0.32) [21]	2.47 (0.24) [127]	0.92	2.66 (0.33) [24]	2.54 (0.20) [37]	2.51 (0.45) [11]	2.57 (0.30) [72]	0.12
3m get up and go, s[a]	Boyd Orr	2.24 (0.23) [50]	2.19 (0.23) [85]	2.25 (0.29) [43]	2.22 (0.25) [178]	0.91	2.25 (0.29) [56]	2.23 (0.28) [111]	2.25 (0.37) [40]	2.24 (0.30) [207]	0.98
3m get up and go, s[a]	CaPS	2.33 (0.24) [256]	2.34 (0.26) [384]	2.31 (0.23) [112]	2.33 (0.25) [752]	0.87	-	-	-	-	-
6m walk, s[a]	LBC1921	1.40 (0.27) [65]	1.42 (0.35) [98]	1.43 (0.24) [49]	1.42 (0.30) [212]	0.57	1.49 (0.25) [93]	1.59 (0.31) [150]	1.51 (0.23) [55]	1.55 (0.28) [298]	0.37
	**Pooled**	**[1268]**	**[1923]**	**[698]**	**[3889]**	**0.39**	**[1179]**	**[1693]**	**[712]**	**[3584]**	**0.25**
Chair rises[b]-10	NSHD	5.39 (1.64) [377]	5.34 (1.90) [609]	5.29 (1.58) [220]	5.34 (1.76) [1206]	0.51	5.08 (1.61) [399]	5.13 (1.58) [566]	4.88 (1.60) [247]	5.06 (1.60) [1212]	0.20
Chair rises[b]-5	ELSA	9.84 (3.19) [702]	9.83 (3.24) [1061]	9.92 (3.35) [392]	9.85 (3.24) [2155]	0.74	9.27 (2.99) [803]	9.44 (3.03) [1206]	9.65 (3.28) [519]	9.43 (3.07) [2528]	0.0268
Chair rises[b]-5	HCS	6.62 (1.42) [199]	6.77 (1.41) [301]	6.84 (1.26) [111]	6.73 (1.39) [611]	0.15	5.84 (1.38) [291]	5.84 (1.47) [422]	6.05 (1.44) [173]	5.88 (1.44) [886]	0.18
Chair rises[b]-5	HAS	5.55 (1.81) [47]	5.57 (1.47) [54]	5.64 (1.17) [19]	5.57 (1.56) [120]	0.86	4.70 (1.22) [22]	5.00 (1.44) [35]	5.79 (1.05) [10]	5.02 (1.35) [67]	0.0439
	**Pooled**	**[1325]**	**[2025]**	**[742]**	**[4092]**	**0.63**	**[1515]**	**[2229]**	**[949]**	**[4693]**	**0.32[c]**
**Standing balance description**	**Cohort**	**RR n (%)[d]**	**RX n (%)[d]**	**XX n (%)[d]**	**Total n (%)[d]**	**p-value**	**RR n (%)[d]**	**RX n (%)[d]**	**XX n (%)[d]**	**Total n (%)[d]**	**p-value**
One-legged- <30s	NSHD	96 (24.7)	140 (22.3)	51 (21.6)	287 (22.9)	0.33	135 (32.4)	184 (31.1)	89 (34.5)	408 (32.2)	0.66
Tandem- <5s	ELSA	89 (10.9)	122 (10.1)	43 (9.4)	254 (10.3)	0.38	150 (15.9)	227 (16.0)	76 (12.7)	453 (15.3)	0.14
Flamingo- <30s	HCS	95 (47.0)	169 (54.3)	65 (56.0)	329 (52.3)	0.09	200 (65.8)	297 (67.7)	118 (66.7)	615 (66.8)	0.78
Flamingo- <30s	Boyd Orr	39 (78.0)	56 (65.9)	31 (73.8)	126 (71.2)	0.60	46 (82.1)	93 (83.8)	26 (65.0)	165 (79.7)	0.07
Flamingo- <30s	CaPS	181 (71.3)	281 (72.4)	80 (67.8)	542 (71.3)	0.63	-	-	-	-	-
	**Pooled**	**[500/1707]**	**[768/2620]**	**[270/968]**	**[1538/5295]**	**0.64**	**[531/1723]**	**[801/2557]**	**[309/1072]**	**[1641/5352]**	**0.47**

a: time taken on logarithmic scale. b: Reciprocal of time taken in sec x 100. c: significant heterogeneity, p-value=0.0241. d: #participants with event (%); Pooled: [#participants with event/total # participants with relevant data]. P-values per X allele. Pooled results based on z-scores. Timed get up and go, chair rises in HAS from Phase II; balance, timed get up and go, chair rises in HCS from both phases.
